# The molecular basis of spectral tuning in blue- and red-shifted flavin-binding fluorescent proteins

**DOI:** 10.1016/j.jbc.2021.100662

**Published:** 2021-04-19

**Authors:** Katrin Röllen, Joachim Granzin, Alina Remeeva, Mehdi D. Davari, Thomas Gensch, Vera V. Nazarenko, Kirill Kovalev, Andrey Bogorodskiy, Valentin Borshchevskiy, Stefanie Hemmer, Ulrich Schwaneberg, Valentin Gordeliy, Karl-Erich Jaeger, Renu Batra-Safferling, Ivan Gushchin, Ulrich Krauss

**Affiliations:** 1 Institut für Molekulare Enzymtechnologie, Heinrich-Heine-Universität Düsseldorf, Forschungszentrum Jülich GmbH, Jülich, Germany; 2 IBI-7: Structural Biochemistry, Forschungszentrum Jülich GmbH, Jülich, Germany; 3 JuStruct: Jülich Center for Structural Biology, Forschungszentrum Jülich, Jülich, Germany; 4 Research Center for Molecular Mechanisms of Aging and Age-Related Diseases, Moscow Institute of Physics and Technology, Dolgoprudny, Russia; 5 Institute of Biotechnology, RWTH Aachen University, Aachen, Germany; 6 IBI-1: Molecular and Cellular Physiology, Forschungszentrum Jülich GmbH, Jülich, Germany; 7 Institut de Biologie Structurale Jean-Pierre Ebel, Université Grenoble Alpes-Commissariat à l’Energie Atomique et aux Energies Alternatives–CNRS, Grenoble, France; 8 Institute of Crystallography, RWTH Aachen University, Aachen, Germany; 9 IBG-1: Biotechnology, Forschungszentrum Jülich GmbH, Jülich, Germany; 10 DWI-Leibniz Institute for Interactive Materials, Aachen, Germany

**Keywords:** flavoprotein, fluorescence, imaging, X-ray crystallography, structure–function flavin-binding fluorescent protein, spectral tuning, photophysics, rational protein design, FbFPs, flavin-binding fluorescent proteins, FMN, flavin mononucleotide, iLOV, improved LOV, IMAC, immobilized metal-ion affinity chromatography, LOV, light, oxygen, voltage, QM/MM, quantum mechanics molecular mechanics

## Abstract

Photoactive biological systems modify the optical properties of their chromophores, known as spectral tuning. Determining the molecular origin of spectral tuning is instrumental for understanding the function and developing applications of these biomolecules. Spectral tuning in flavin-binding fluorescent proteins (FbFPs), an emerging class of fluorescent reporters, is limited by their dependency on protein-bound flavins, whose structure and hence electronic properties cannot be altered by mutation. A blue-shifted variant of the plant-derived improved light, oxygen, voltage FbFP has been created by introducing a lysine within the flavin-binding pocket, but the molecular basis of this shift remains unconfirmed. We here structurally characterize the blue-shifted improved light, oxygen, voltage variant and construct a new blue-shifted CagFbFP protein by introducing an analogous mutation. X-ray structures of both proteins reveal displacement of the lysine away from the chromophore and opening up of the structure as instrumental for the blue shift. Site saturation mutagenesis and high-throughput screening yielded a red-shifted variant, and structural analysis revealed that the lysine side chain of the blue-shifted variant is stabilized close to the flavin by a secondary mutation, accounting for the red shift. Thus, a single additional mutation in a blue-shifted variant is sufficient to generate a red-shifted FbFP. Using spectroscopy, X-ray crystallography, and quantum mechanics molecular mechanics calculations, we provide a firm structural and functional understanding of spectral tuning in FbFPs. We also show that the identified blue- and red-shifted variants allow for two-color microscopy based on spectral separation. In summary, the generated blue- and red-shifted variants represent promising new tools for application in life sciences.

Flavin-binding fluorescent proteins (FbFPs), engineered from prokaryotic and eukaryotic light, oxygen, voltage (LOV) photoreceptors (1, 2), are promising fluorescent reporter proteins. Their small size (about 10 kDa for monomeric FbFPs such as the improved LOV (iLOV) protein derived from *Arabidopsis thaliana* phototropin photoreceptors (1)) and their oxygen-independent fluorescence make them ideal reporters to monitor transport (1, 3) and anaerobic biological processes (4, 5, 6, 7). They are also used as the donor domain in FRET-based biosensors, for example, for molecular oxygen or pH (8, 9). Proteins from the GFP family are very popular for similar applications in life sciences but have clear disadvantages because of their larger size and their need of sufficient molecular oxygen for chromophore maturation, which often causes failure when used under anaerobic conditions. Moreover, FbFPs (1, 2), LOV photosensory domains, and other flavin-binding photoreceptors (summarized in (10, 11)) have been utilized as biosensors (8, 9, 12), photosensitizers (13, 14, 15), for example, correlative light electron microscopy (15), chromophore-assisted light inactivation (16), and/or as optogenetic tools for the reversible control of biological processes (recently reviewed (10)).

Applications such as multicolor imaging, design of solely FbFP-based FRET biosensors, multicolor correlative light electron microscopy, chromophore-assisted light inactivation, and LOV-based optogenetics would require development of spectrally shifted FbFPs or LOV domains. In contrast to GFP-like fluorescent reporter proteins, whose absorption and fluorescence maxima can be tuned by exchanging certain chromophore-constituting amino acids (17), the absorption and fluorescence of FbFPs and other flavoproteins depends on noncovalently bound flavins, whose structure cannot be influenced by mutation. This is a severe drawback of FbFPs, which has so far limited all color-tuning efforts for this important new family of fluorescent reporters.

At present, only blue-shifted FbFPs are available, derived by substituting a fully conserved glutamine (Q489 in iLOV) against valine (18) or lysine (19). For the latter case, molecular dynamics (MD) simulations and quantum mechanics molecular mechanics (QM/MM) calculations previously suggested that the introduced lysine (K489 in iLOV–Q489K) flips away from the chromophore, which accounts in theory for the observed blue shift (19). This hypothesis, which was solely based on simulations of an *in silico*–generated iLOV–Q489K model, remains structurally unproven. Likewise, theoretical predictions suggested that the retention of a charged amino acid, such as lysine, close to the flavin mononucleotide (FMN) ring system should result in red-shifted spectral properties (19, 20, 21), which so far could not be achieved experimentally. In addition, the transferability of the proposed mutations and mechanisms for spectral tuning have so far not been validated for FbFPs other than iLOV, which however is a prerequisite for widespread implementation.

## Results and discussion

**Figure 1:**
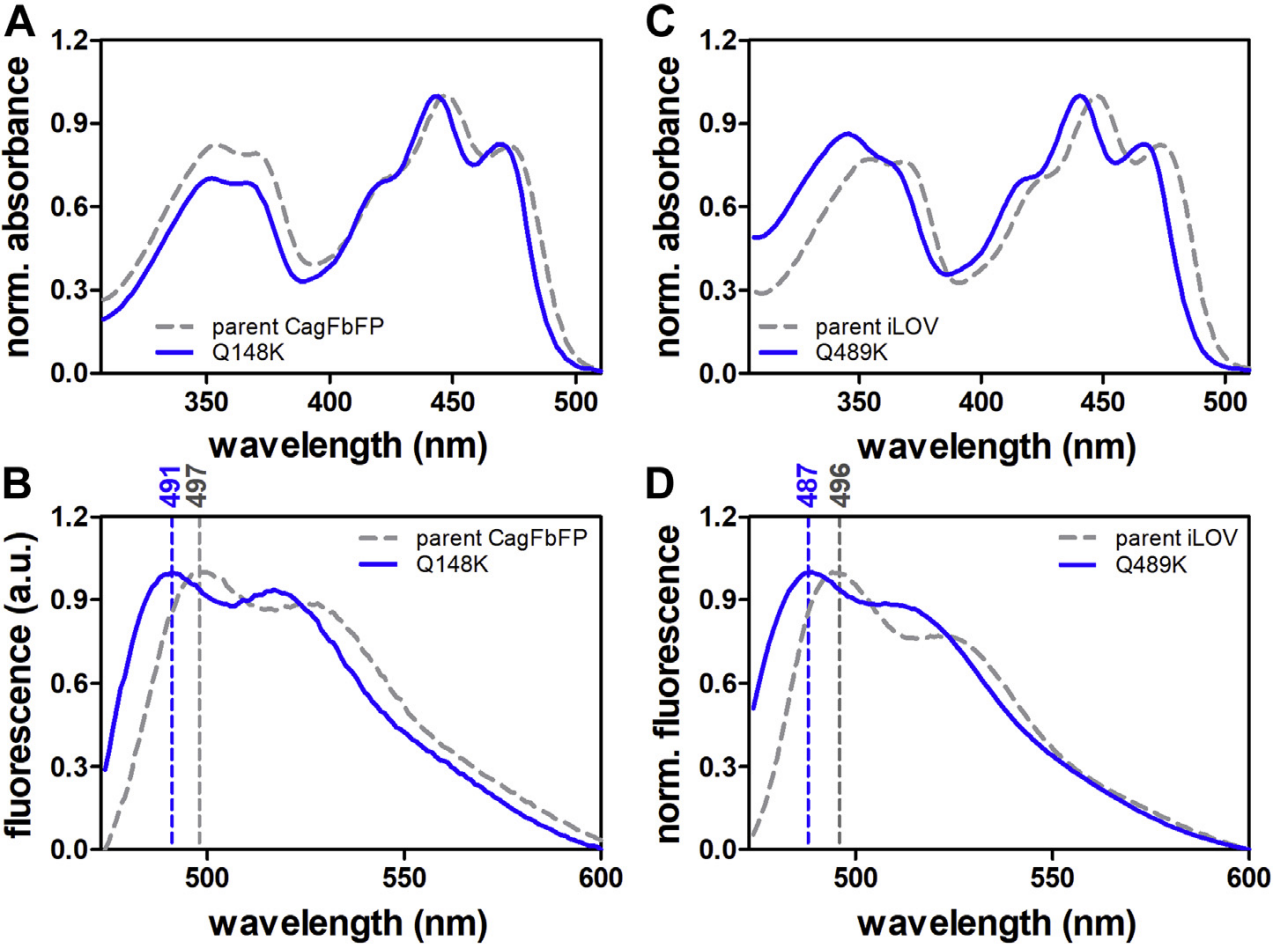
**Spectral properties of blue-shifted FbFPs.** Absorption (*A* and *C*) and fluorescence emission spectra (*B* and *D*) of parental CagFbFP and iLOV (*gray*, *dashed line*) and their *blue-shifted* CagFbFP–Q148K/iLOV–Q489K variants (*blue*, *solid line*). *Dashed lines* of the respective color mark fluorescence emission maxima. All spectra are normalized to the corresponding maximum. FbFPs, flavin-binding fluorescent proteins.

To verify the transferability of the blue-shifting Q489K mutation, identified for the plant FbFP iLOV (19), we introduced the corresponding Q148K mutation into the recently presented thermostable CagFbFP protein from thermophilic phototrophic bacterium *Chloroflexus aggregans* (22). In contrast to the monomeric, plant-derived iLOV protein, the bacterial CagFbFP protein is a dimer in solution (22). In terms of sequence, iLOV and CagFbFP share only about 40% identical amino acid positions, highlighting the distinct origin of CagFbFP. With regard to its flavin-binding site and the overall LOV domain structure, iLOV and CagFbFP are, however, very similar (C-α atom RMSD 0.58 Å over 104 aligned residues) (22). To study the spectral properties of the CagFbFP–Q148K variant, we heterologously produced the variant in *Escherichia coli* and purified it to homogeneity. The absorption and fluorescence emission spectra of CagFbFP–Q148K are shown in Figure 1, *A* and *B*. For comparison, the corresponding spectra of iLOV–Q489K are depicted in Figure 1, *C* and *D*.

Much like iLOV–Q489K (Fig. 1, *C* and *D*; Table 1), CagFbFP–Q148K possesses a blue-shifted S_0_→S_1_ absorption band with a maximum at around 444 nm (≈3 nm blue-shifted relative to parental CagFbFP) and a blue-shifted fluorescence-emission maximum (≈6 nm blue shift relative to parental CagFbFP; Fig. 1, *A* and *B*; compare the gray dashed line and solid blue line; Table 1). Thus, the spectral effects previously seen in the plant-derived, monomeric iLOV–Q489K protein are well reproduced in the thermostable, dimeric CagFbFP protein of bacterial origin, thus hinting at a conserved structural mechanism.

**Table 1:** Photophysical properties of all iLOV and CagFbFP variants studied here

Protein	λ_max-absorption_ (nm)	λ_max-emission_ (nm)	Shift (nm)^a^	*Ф*_*F*_	*τ*_*av*_/ns
iLOV	447	496	-	0.33 ± 0.01	4.47 ± 0.03
iLOV–Q489K	441	487	−9	0.35 ± 0.01	4.03 ± 0.08
iLOV–Q489K–V392T	447	502	+6	0.33 ± 0.02	4.48 ± 0.03
CagFbFP	447	497	-	0.36 ± 0.01^b^	4.53 ± 0.03
CagFbFP–Q148K	444	491	−6	0.24 ± 0.01	3.28 ± 0.06
CagFbFP–Q148K/I52T	450	504	+7	0.27 ± 0.01	3.82 ± 0.05
CagFbFP–I52T	450	497	0	0.38 ± 0.06	4.42 ± 0.07

a Relative to emission maximum of the corresponding parental protein.

b Data present in (22).

**Figure 2:**
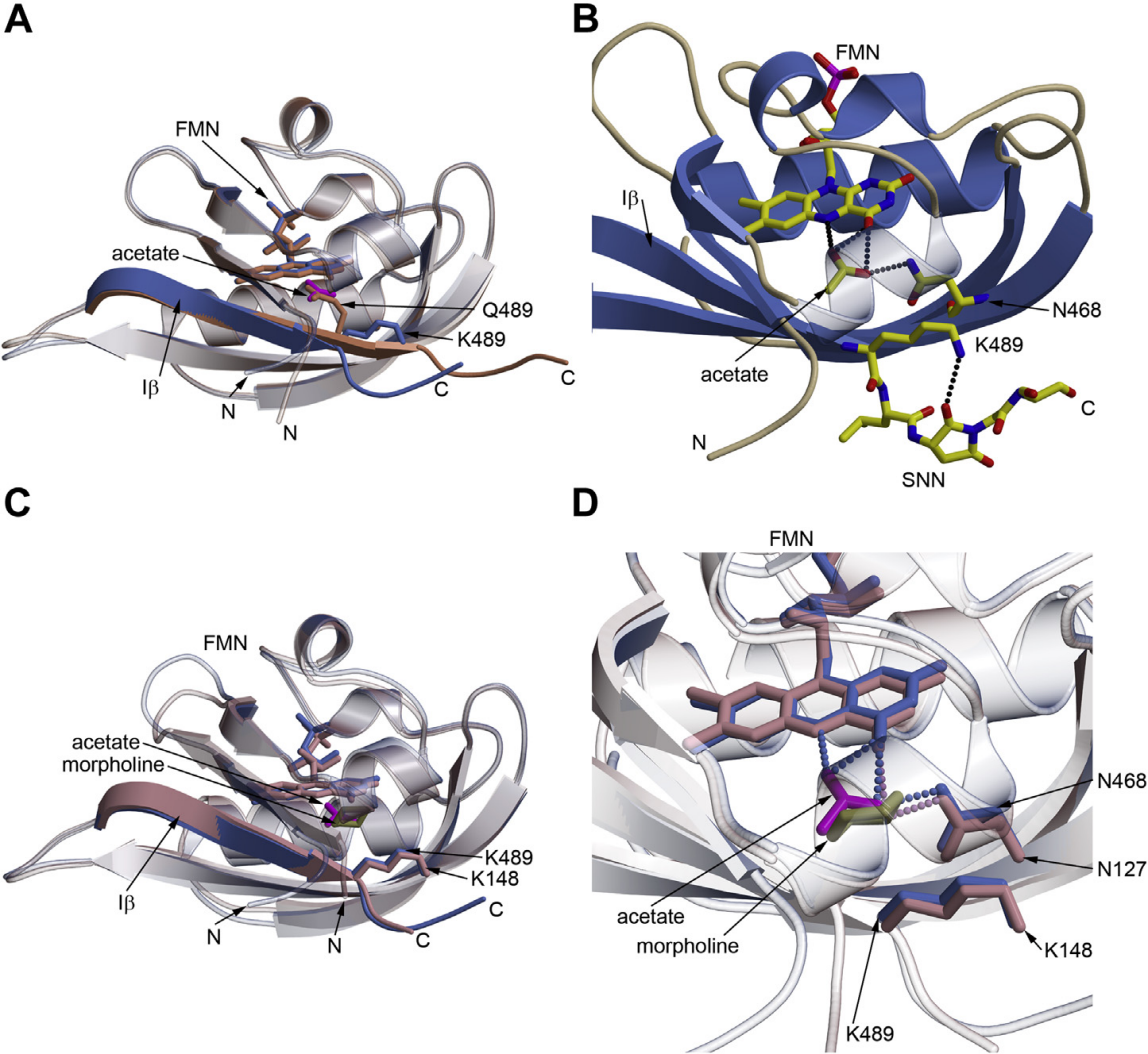
**Structural comparison of parental iLOV, iLOV–Q489K, and CagFbFP–Q148K.***A*, superposition of parental iLOV (PDB ID: 4EES; *light salmon*) with iLOV–Q489K (*cornflower blue*). Introduction of Q489K results in shortening of the C-terminal Iβ strand and makes it bend downward. A buffer molecule fragment [CH_3_COO]^−^ (acetate anion, *magenta*) occupies the position of the side chain of Q489 in parental iLOV. *B*, structure of iLOV–Q489K. Depicted are the acetate anion with its hydrogen bond network to FMN and N468 (as a *stick model* in standard atomic coloration). The introduced K489 interacts with the modified C terminus, formed by deamidation of N491 and G492, yielding a succinimide intermediate (SNN L-3-aminosuccinimide). *C*, superposition of iLOV–Q489K (*cornflower blue*) and CagFbFP–Q148K (*plum*). Both structures show the same rotamer conformation of the lysine side chain, as well as a bending of the backbone. In addition, the respective buffer molecules of iLOV–Q489K and CagFbFP–Q148K are shown, namely the acetate ion (*magenta*) and the morpholine [O(CH_2_CH_2_)_2_NH] (*olive green*). The conformation of the Aβ–Bβ loop is different between iLOV–Q489K and CagFbFP–Q148K (*upper left part* of Fig. 2*C*). However, because the same overall difference is also seen between parent iLOV and CagFbFP, this difference is not a consequence of the introduced mutation. *D*, environment of the buffer molecules found in iLOV–Q489K and CagFbFP–Q148K, namely the acetate anion and the morpholine molecule, in an almost identical position. Both molecules form hydrogen bonds with the FMN, N468 (iLOV–Q489K), or N127 (CagFbFP–Q148K), respectively. Color coding as in panel *C*. *Dashed lines* represent hydrogen bonds with a donor–acceptor distance of ≤3.2 Å.

To elucidate the structural basis for the blue-shifted spectral properties, we determined the structure of iLOV–Q489K. Tetragonal crystals (space group P4_3_2_1_2; one molecule per asymmetric unit) grew within 6 weeks and diffracted up to 1.45 Å resolution. Data and refinement statistics are summarized in Table S1. Globally, the iLOV–Q489K structure is very similar to the structure of parental iLOV (PDB ID: 4EES; RMSD is 0.69 Å over the C-α atoms of 104 aligned residues) showing a typical α+β Per-Arnt-Sim domain topology with the secondary structure elements in the order Aβ-Bβ-Cα-Dα-Eα-Fα-Gβ-Hβ-Iβ. Please note that, the two available parental iLOV structures 4EES (one molecule in the asymmetric unit) and 4EET (two molecules in the asymmetric unit) show a relatively high RMSD value of 0.93 Å for all residues (including the termini), which reduces to 0.55 Å for the LOV core domain only (residues 389–489). Surprisingly, the iLOV–Q489K and parental iLOV structures diverge at the C-terminal end following the introduced lysine (K489 in iLOV–Q489K) (Fig. 2*A*).

Although in parental iLOV the Iβ-strand is constituted by the residues 482 to 491, Iβ is shortened by two residues in iLOV–Q489K and ends at K489, followed by a kink in the subsequent residue, with the C terminus pointing away from the core compared with the parental iLOV (Fig. 2*A*). In addition, the C-terminal end of Iβ becomes detached from the core (in the following described as unlatching), and the last three C-terminal residues lack detectable density. Interestingly, D491 and G492 have undergone post-translational modification to yield a cyclic imide structure (succinimide), which we speculate to be an artifact of protein aging (23). Nonenzymatic intramolecular reactions can result in deamidation-dependent isomerization, and racemization of protein and peptide asparaginyl and aspartyl residues *via* succinimide intermediates. The rate of deamidation reaction in proteins depends on multiple factors, including the primary sequence, higher-order structure, pH, temperature, and components in the solution. The Asn–Gly is known to be the most common potential deamidation site in proteins (23, 24, 25). In parental iLOV, Q489 forms an H-bond with the FMN-O4 atom. In contrast, in the K489 mutant, the introduced K489 extends away from FMN and forms an H-bond with the O2 atom of the succinimide ring (K489-NZ^…^SNN-O2, 3.1 Å) (Fig. 2*B*).

Collectively, this results in opening up of the LOV core domain (Fig. 2, *A* and *B*), which is expected to result in the influx of solvent molecules. In fact, additional electron density is present close to FMN-N5, which we interpret as an acetate ion (Fig. 2*B*, Fig. S1*A*), likely derived from the crystallization screen, which contained acetate buffer. Interestingly, in iLOV–Q489K, this ion occupies the former position of the Q489 side chain of parental iLOV. The K489 conformation, experimentally observed here, is distinct from the ones observed in previous MD simulations, suggesting that the previously described MD-identified K489_out_ conformation (19, 20) represents a local energy minimum.

Aside from those differences, the introduced lysine adopts a conformation that is facing away from the chromophore, which, in light of previous QM/MM calculations (19, 20), accounts for the blue-shifted spectral properties of the variant, that is, due to the absence of a direct H-bonding interaction between the flavin and the introduced Lys. This is corroborated by the observation that the introduction of hydrophobic residues at the same position is known to also result in blue-shifted spectral properties (18). Increased solvent influx, as observed in our structures, might result in a reduced magnitude of the blue shift because of the stabilization of solvent/buffer molecules that interact with the flavin (similarly to the Q489 side chain in parental iLOV).

To verify that the structural changes, which we observed for iLOV–Q489K, are indeed a consequence of the introduced Q489K mutation and are not due to the observed succinimide modification, we obtained the structure of the corresponding CagFbFP–Q148K variant. Altogether, we were able to obtain three types of CagFbFP–Q148K crystals, all using the precipitant solutions with pH of 6.5 (data and refinement statistics are summarized in Table S1). In all of the obtained models, the C terminus of the protein is clearly unlatched, and the side chain of the introduced lysine is facing away from FMN as was found in iLOV–Q489K (Fig. 2*C*). Thus, the succinimide modification present in iLOV–Q489K does not influence the backbone and the conformation of the introduced Lys because this modification is absent in all CagFbFP–Q148K structures. Interestingly, the highest-resolution structure (PDB ID: 6YX4, determined at a resolution of 1.36 Å; C-α atom RMSD to iLOV–Q489K of 0.77 Å for 102 aligned residues) revealed a six-membered ring in a chair conformation close to the FMN (Fig. 2*D*; Fig. S1*B*), which we speculate to derive from 2-(N-morpholino)ethanesulfonic acid present in the precipitant solution. Interestingly, the protein also crystallized using another precipitant solution for which we obtained crystals in two crystal forms. P2_1_2_1_2 crystals (PDB ID: 6YX6) diffracted anisotropically, and we applied ellipsoidal truncation (see Supporting Materials and Methods for details) using the resolution cut-offs of 1.5 and 2.05 Å. Despite having been crystallized under different conditions and in a different space group, all alternative CagFbFP–Q148K structures showed an extended outward-facing K148 conformation and an unlatched C terminus relative to parental iLOV (exemplarily shown for the highest resolution structure, PDB ID: 6YX4; Fig. 2, *C* and *D*). Therefore, we conclude that irrespective of the buffer and post-translational modification, the C terminus of the iLOV–Q489K and CagFbFP–Q148K variants is unlatched, the LOV core domain is opened up to allow solvent ingress, and the introduced lysine occupies an extended conformation facing away from the FMN chromophore. The related variant CagFbFP–Q148R, which was recently reported, also shows blue-shifted spectral properties, featuring an unlatched C terminus (26). We thus suggest these structural changes as the characteristic features for a blue-shifted absorption and emission spectrum, that is, when compared with the parental FbFPs.

**Figure 3:**
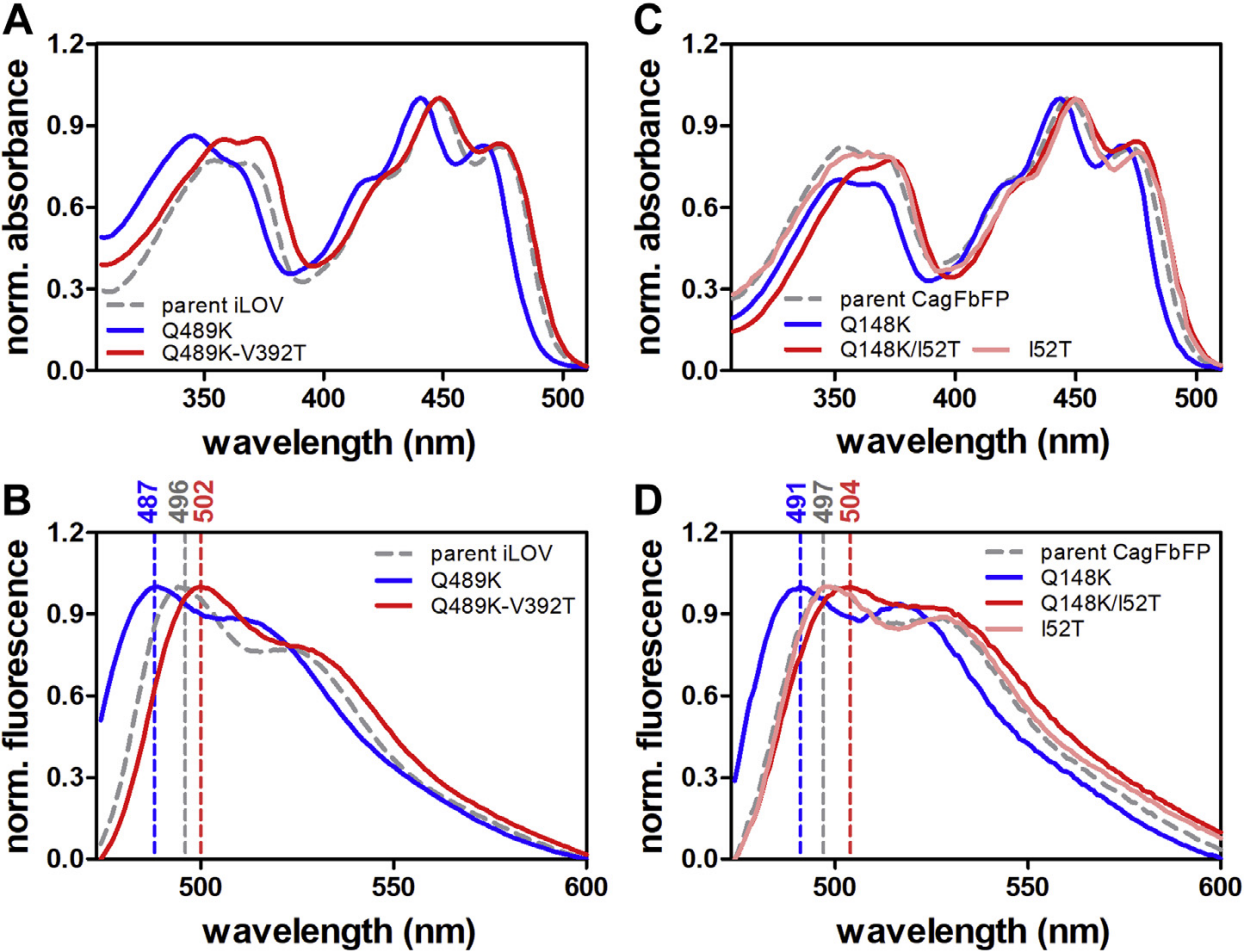
**Spectroscopic characterization of iLOV and CagFbFP variants.** Absorption (*A* and *C*) and fluorescence-emission spectra (*B* and *D*) of iLOV and its variants (*A* and *B*) and CagFbFP and its variants (*C* and *D*). The spectra of the respective parental proteins are shown as *gray dashed lines*, the *blue-shifted* variants (iLOV–Q489K; CagFbFP–Q148K) with *blue solid lines* and the red-shifted variants (iLOV–Q489K–V392T; CagFbFP–Q148K–I52T) with *red solid lines*. In addition, the absorption and fluorescence-emission spectra of CagFbFP–I52T are shown as *light-red solid lines*. *Dashed lines* of the respective color mark fluorescence emission maxima. All spectra are normalized to the corresponding maximum.

As shown by our iLOV–Q489K and CagFbFP–Q148K structures presented previously, introduction of the single Q489K/Q148K substitution results in blue-shifted spectra, likely because of the displacement of the introduced lysine away from the FMN chromophore. In contrast, previous computational studies have suggested that retention of a charged lysine close to the chromophore should result in red-shifted spectral properties (19, 21). We therefore reasoned that secondary mutations are needed to stabilize the introduced lysine to stay close to the FMN ring system. Based on our iLOV–Q489K structure, we selected G487 and V392 as suitable positions as they are close to the introduced K489 (Fig. S1*C*), so that side-chain alterations at those sites could stabilize K489 to retain its position close to FMN. Following this rationale, we set out to mutate G487 and V392 in iLOV–Q489K. To sample a larger sequence space and to increase the chances for identifying a red-shifted FbFP variant, we generated two site-saturation libraries of iLOV–Q489K. Overall, 465 clones were screened per library. The fluorescence-emission maxima of a subset of promising variants identified by the screen are given in Table S2. Although saturation mutagenesis at position G487 yielded no variants with red-shifted emission relative to parental iLOV, two variants with red-shifted emission maxima relative to iLOV–Q489K (Table S2, Fig. S2) were identified. Both variants turned out to contain a serine substitution at position G487 (Table S2). Site saturation at position V392 yielded more promising results as overall 16 variants with red-shifted spectral properties could be identified (Table S2). 14 of 16 variants carried a threonine at position 392, whereas two variants contained either a cysteine or an alanine (Table S2). We selected the iLOV–V392T–Q489K variant, with the largest red shift relative to parental iLOV (variant B5-B12; Table S2, 8 nm red shifted fluorescence emission), for further studies, and overexpressed and purified the protein. Absorption and fluorescence emission spectra of the purified protein are shown in Figure 3*A*. Compared with parental iLOV (Fig. 3, *A* and *B*; gray dashed line), the double mutant iLOV–V392T–Q489K possesses a very similar absorption maximum at around 447 nm (Fig. 3*A*; red solid line, Table 1). The main fluorescence-emission maximum is red shifted by 6 nm to 502 nm (Fig. 3*B*; red solid line; Table 1). Compared with iLOV–Q489K (Fig. 3, *A* and *B*; blue solid line), the double variant shows a red-shifted absorption maximum and a fluorescence-emission maximum shifted by 15 nm (Fig. 3, *A* and *B*; red solid line; Table 1). To verify the transferability of the mutation, we introduced the corresponding I52T mutation into CagFbFP–Q148K. The resulting purified protein showed very similar spectral properties as iLOV–V392T–Q489K, that is, basically no shift in the absorption maximum relative to parental CagFbFP (Fig. 3*C*; compare solid red line and gray dashed line; Table 1) and a 7-nm red-shifted fluorescence-emission maximum (Fig. 3*D*; compare gray dashed line and solid red line; Table 1). To rule out that the I52T mutation alone is sufficient to generate the observed red-shift, we introduced the I52T mutation in parental CagFbFP (Fig. 3, *C* and *D*; light red solid line; Table 1). The resulting variant showed nearly identical spectral properties as parental CagFbFP (Fig. 3, *C* and *D*; gray dashed line; Table 1). The latter is corroborated by the X-ray structure of CagFbFP-I52T (see Table S1 for data and refinement statistics), which is virtually identical to parental CagFbFP (C-α atom RMSD 0.52 Å, 102 aligned residues). Most importantly, it does not show an unlatched C terminus as observed for CagFbFP–Q148K (Fig. S1*D*).

We thus conclude that a single secondary mutation in blue-shifted iLOV–Q489K and CagFbFP–Q148K, which places a polar threonine residue into the flavin-binding pocket, is sufficient to invert the spectral properties from blue- to red-shifted relative to the parent protein. To illustrate why the FbFP variants, identified here, were not identified by random mutagenesis or gene shuffling in various mutagenesis campaigns (1, 27, 28) until now, we estimated the probability to identify the iLOV–V392T–Q489K from a large random error-prone PCR library. Here, when screening an error-prone PCR library having 10^6^ variants, generated with a medium mutation rate of 8 mutations/kb (29), the probability of discovering at least one copy of iLOV–V392T–Q148K variant is approximately 0.0014. This assumes a roughly uniform mutation spectrum and independence across variants and DNA positions. Please note that for screening of 10^6^ clones, ultrahigh-throughput systems, such as FACS-sorting or droplet-based microfluidics screens (30), would be needed. Hence, ultrahigh-throughput methods with sufficient sensitivity to differentiate between blue- and red-shifted variants, as well as mutagenesis methods that, for example, allow the parallel saturation of multiple sites (31, 32, 33), or high-error rate error-prone PCR methods (34), would likely be needed for further color-tuning of FbFPs.

**Figure 4:**
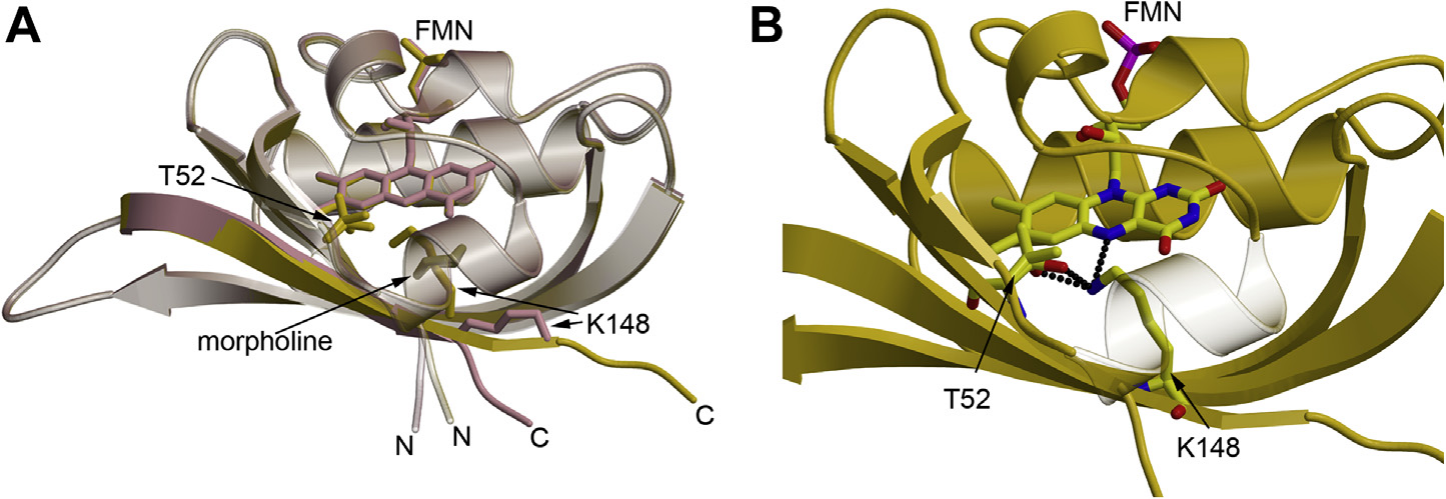
**Structure of CagFbFP–I52T–Q148K.***A*, superposition of CagFbFP–Q148K (*plum*) with CagFbFP–I52T–Q148K (*gold*). In addition, the introduced K148 and T52 (with two rotamer conformations) are depicted. *B*, structure of CagFbFP–I52T–Q148K with hydrogen bonds between K148, T52, and the FMN. T52 shows two clear conformations; this is conditionally also to be observed for lysine, but for reasons of clarity, only one conformation is shown. Additional details and the corresponding electron density maps are shown in Fig. S3. *Dashed lines* represent hydrogen bonds with a donor–acceptor distance of ≤3.2 Å. FMN, flavin mononucleotide.

To validate our design approach and elucidate the structural basis for the red-shifted spectral properties of the corresponding iLOV and CagFbFP variants, we set up crystallization trials of both variants. We readily obtained orthorhombic crystals for CagFbFP–I52T–Q148K (space group P2_1_2_1_2, two molecules per asymmetric units) that diffracted up to 1.8 Å. Compared with the blue-shifted iLOV–Q489K and CagFbFP–Q148K variants, the red-shifted double variant shows a closed FMN binding pocket with no unlatched C terminus (Fig. 4*A*; C-α atom core RMSD: 0.48 Å with 102 aligned residues).

The introduced threonine (T52) shows two conformations in both subunits, one with the side-chain oxygen facing toward G146 and a second one with the side-chain oxygen facing toward F69 (approximate occupancies of the two conformations are 65% and 35%, respectively). K489 adopts one major conformation in both subunits (approximate occupancy 65%) in which the side chain extends toward the introduced T52 and the FMN moiety. Interactions between both threonine conformations and the lysine are feasible with interatomic distances between the lysine NZ atom and the threonine side-chain oxygen of 2.5 Å or 2.6 Å in both subunits (Fig. 4*B*). It is remarkable that the double mutation (CagFbFP–I52T–Q148K) forces the lysine into a new conformation by hydrogen bonding with threonine, thus preventing the bending of the C terminus, which restores a nearly “native” closed LOV-core conformation.

In chain A, the introduced lysine adopts a second conformation with its side chain facing away from T52 pointing instead toward N127 and F125 (occupancy 35%) (Fig. S3). This indicates that both of the introduced residues show a certain degree of flexibility. Stressing this notion, other residues such as D55, Q112, R119, D121, Q123, Q128, S130, S132, and V145 show multiple conformations, and two backbone conformations are observed in chain B for residues following V147. In conclusion, the CagFbFP–I52T–Q148K structure obtained here is in full agreement with our design concept, with the introduced threonine stabilizing the lysine in a conformation close to the FMN-N5 atom (interatomic distances 3.2 Å and 3.0 Å in chains A and B, respectively) (Fig. 3*B*).

To gain a better understanding of the photophysics of the different variants, we determined their fluorescence quantum yields *Ф*_*F*_ and fluorescence lifetimes *τ*_*av*_ (Table 1). The corresponding fluorescence decays showed biexponential decay behavior and are shown in Figs. S4 and S5, respectively. The first unexpected finding is that the photophysical parameters of the two parental proteins, CagFbFP and iLOV, are almost identical. Absorption and emission maxima, fluorescence quantum yields, and fluorescence lifetimes are virtually identical (Table 1). This is quite remarkable because origin and amino acid sequence of the two proteins are rather different and *Ф*_*F*_ and *τ*_*av*_ of FbFPs are known to vary by more than 50% (18).

The photophysical properties of the CagFbFP variants are relatively straightforward to interpret. Compared with the parental CagFbFP protein (*Ф*_*F*_ = 0.35 ± 0.01; *τ*_*av*_ = 4.53 ± 0.03 ns), we observed a significant reduction of *Ф*_*F*_ along with similarly shorter *τ*_*av*_ for CagFbFP–Q148K (*Ф*_*F*_ = 0.24 ± 0.01; *τ*_*av*_ = 3.28 ± 0.03 ns), which can be attributed to dynamic quenching processes by solvent molecules, that is, due to increased solvent access, as revealed by our structural analyses (see above). This scenario is corroborated by the data obtained for CagFbFP–I52T, which is structurally identical to the parent protein and showed, within the experimental error, very similar *Ф*_*F*_ and *τ*_*av*_ values (*Ф*_*F*_ = 0.38 ± 0.06; *τ*_*av*_ = 4.42 ± 0.07 ns). The same holds for CagFbFP–I52T–Q148K, which possesses a closed FMN-binding pocket with K148 being in H-bonding distance to the FMN-N5 atom, and consequently shows increased *Ф*_*F*_ and *τ*_*av*_, (*Ф*_*F*_ = 0.27 ± 0.01; *τ*_*av*_ = 3.82 ± 0.03 ns) relative to CagFbFP–Q148K. Please note, however, that CagFbFP–I52T–Q148K does not show a completely parental CagFbFP–like *τ*_*av*_ value, hinting at a certain degree of flexibility or conformational heterogeneity in this variant. This is indeed observed in the CagFbFP–I52T–Q148K structure, which features multiple conformations for I52 and K148 as well as multiple backbone conformations for the C terminus in chain A (see above).

Surprisingly, although having very similar spectral properties and overall structures, the situation is less clear for iLOV and its variants. Compared with parental iLOV (*Ф*_*F*_ = 0.33 ± 0.01), iLOV–Q489K possesses a very similar *Ф*_*F*_ (*Ф*_*F*_ = 0.35 ± 0.01), whereas *τ*_*av*_ values for iLOV–Q489K are shorter (*τ*_*av*_ = 4.03 ± 0.08 ns) than that of the parental protein (*τ*_*av*_ = 4.47 ± 0.08 ns). This trend cannot be explained by dynamic quenching processes, which occur in the excited state and hence influence both *Ф*_*F*_ and *τ*_*av*_ in a similar fashion. To complicate matters, similar to the situation for the CagFbFP–I52T–Q148K variant, the corresponding iLOV–V392T–Q148K variant possesses virtually identical *Ф*_*F*_ and *τ*_*av*_ (*Ф*_*F*_ = 0.33 ± 0.02; *τ*_*av*_ = 4.48 ± 0.03 ns) as the parent protein. Thus, either the iLOV–Q489K variant or the parent iLOV protein shows divergent behaviors as compared with the parent CagFbFP protein. Possible explanations include a change in the electronic structure of the first excited singlet state (perhaps also the ground state) of iLOV–Q489K, which manifests in blue-shifted absorption and emission spectra and an in total faster deactivation of the first excited singlet state with larger relative contribution from fluorescence. In addition, altered photochemical properties, for example, increased light-dependent formation of the neutral FMN semiquinone radical FMNH^•^ (35) in the parent protein or—more simple—an increase in the rate of internal conversion relative to iLOV–Q489K, and concomitantly, a decrease in *Ф*_*F*_, could also account for the discrepancy between the iLOV variants and their CagFbFP counterparts.

To computationally probe the hypothesis that the experimentally observed lysine conformations of the blue- and red-shifted variants are the cause for the observed spectral properties, we performed QM/MM calculations based on the corresponding crystal structures. From the calculated vertical excitation energies, we observed that the calculated absorption and emission wavelengths follow the same general trend, that is, with regard to the observed direction of the spectral shift, as the experimental ones (Table S3). Our calculations thus support the idea that the Q489K/Q148K substitutions, and hence the resulting structural changes (Iβ unlatching) in iLOV and CagFbFP, are the cause for blue-shifted absorption and fluorescence emission with respect to the parental proteins. The double substitutions (Q489K/Q148K and V392T/I52T) in the two proteins in turn lead to red-shifted absorption and fluorescence emission by stabilizing the introduced lysine in a position close to the FMN chromophore.

**Figure 5:**
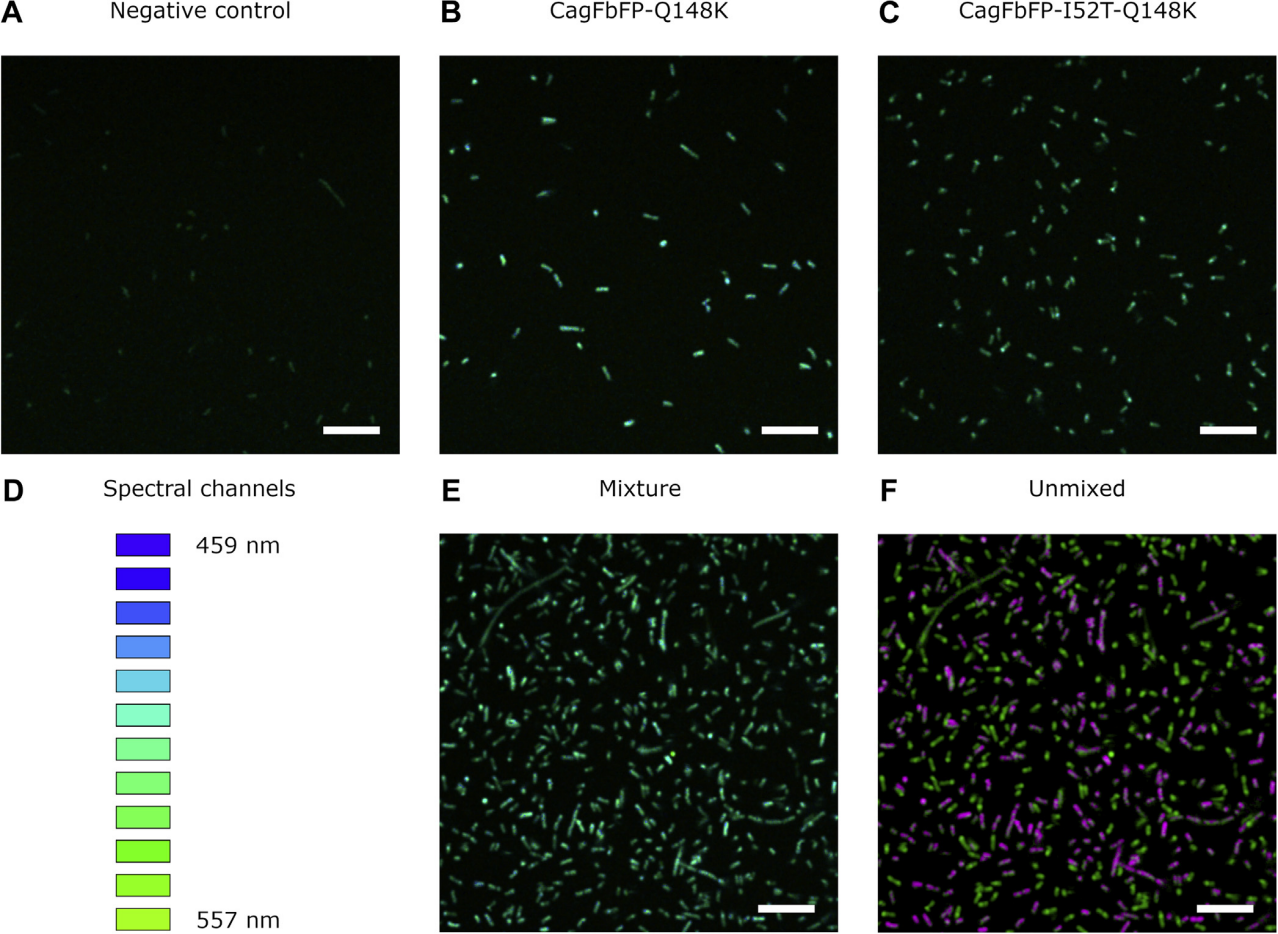
**Imaging of *Escherichia coli* cells transformed with different FbFP variants.** Combined fluorescence intensity and fluorescence spectrum representation of the emission in the pixels is shown. *A*, untransformed *E. coli* cells used as the negative control. *B*, *E. coli* cells expressing CagFbFP–Q148K. *C*, *E. coli* cells expressing CagFbFP–I52T–Q148K. *D*, diagram showing the perceived colors for different fluorescence acquisition channels. Twelve channels from 459 to 557 nm with a 9-nm step were used. *E*, mixture of *E. coli* cells expressing CagFbFP–Q148K and *E. coli* cells expressing CagFbFP–I52T–Q148K. *F*, the same as those shown in panel *E* processed using linear unmixing. The cells expressing CagFbFP–Q148K are highlighted in *magenta*, and the cells expressing CagFbFP–I52T–Q148K are highlighted in *green*. All images except for those in panel *F* were obtained using the same hardware and software settings. The scale bar represents 10 μm. FbFPs, flavin-binding fluorescent proteins.

In many physiology and cell biology studies, it is necessary to observe simultaneously many proteins and cell parameters, and the number of spectrally different detection channels is often a limiting factor. In commercial fluorescence microscopes, two, three, or four spectrally resolved channels are available. The new FbFP variants show similar but significantly different fluorescence spectra and fluorescence lifetimes, which can be exploited in fluorescence microscopy applications utilizing the same excitation wavelength and emission detection channel. A separation of two FbFPs by spectrum or lifetime difference excited with the same laser and observed in the same detection channel adds an additional detection channel, for example, one more protein can be observed. We wanted to demonstrate the usefulness of the new FbFP mutants for this purpose using two of them and chose a blue- and red-shifted FbFP mutant as an example. To this end, we transformed the *E. coli* cells with plasmids carrying the genes for CagFbFP–Q148K (blue-shifted variant) and CagFbFP–I52T–Q148K (red-shifted variant). Compared with untransformed cells, both new strains revealed bright fluorescence (Fig. 5, *A*–*D*). When mixed, the cells of the two strains—although not unequivocally distinguishable in the fluorescence intensity image (even with the spectral dimension used in the image presentation)—can clearly be differentiated from each other using spectral unmixing (Fig. 5, *E* and *F*). Therefore, the identified mutants allow for two-color microscopy with a single excitation wavelength and observed in the same spectral range. Instead of distinguishing different bacterial cells, also different cell organelles or different proteins in mammalian cells could be distinguished.

## Conclusions

The presented work provides a firm structural understanding of spectral tuning in FbFPs, an emerging class of fluorescent reporters. Simultaneously, the presented data improve our understanding of the photophysics and the tunability of the spectral properties for flavins in general, which are essential cofactors in a variety of enzymatic systems and various photoreceptor families. Finally, the generated blue- and red-shifted variants represent promising fluorescent reporters with photophysical properties similar to their respective parent proteins and hence should find application as oxygen-independent reporters in life sciences. Although the blue- and red-shifted variants are spectrally not very different, differentiation between the two proteins is possible in combination with modern fluorescence techniques such as spectral unmixing, a commercially available technique with high-end confocal fluorescence microscopes. This combination opens up the possibility of multicolor imaging in anaerobic settings, where GFP and related proteins do not work because of their oxygen-dependent fluorophore maturation (2, 4, 5). In addition, the large fluorescence lifetime difference between CagFbFP and its Q148K variant allows similar differentiation of two bacteria or proteins by fluorescence lifetime imaging with only one excitation wavelength.

## Experimental procedures

### Cloning and site-directed and site-saturation mutagenesis of iLOV

Site saturation at position 392 and 487 of the iLOV encoding gene (*Arabidopsis thaliana*, Phot1 numbering) was achieved by two single-primer reactions performed in parallel, which were, after initial amplification, combined to allow site saturation using a QuikChange PCR approach (36). Oligonucleotide primers were designed using a degenerate NNS codon instead of the parental target codon. The initial two parallel reactions contained pET28a–iLOV–Q489K (19) as a template with 0.5 μM iLOV-V392X_fw: 5′- GC CAT ATG ATA GAG AAG AAT TTC NNS ATC ACT GAT CCT AGG CTT CCC GA -3’ and iLOV-V392X_rev: 5′- T CGG GAA GCC TAG GAT CAGT GAT SNN GAA ATT CTT CTC TAT CAT ATG GC -3′ or the corresponding iLOV-G487X_fw: 5′- G GGA GAG CTT CAA TAC TTC ATC NNS GTG AAA CTC GAT GGA AGT GAT C -3′ and iLOV-G487X_rev: 5′- G ATC ACT TCC ATC GAG TTT CAC SNN GAT GAA GTA TTG AAG CTC TCC C -3′ primer, respectively, 0.2 mM dNTPs, 1x GC-buffer and Phusion High-Fidelity DNA polymerase (Thermo Fisher Scientific). Amplification was achieved using the following protocol: initial denaturation for 3 min at 98 °C, followed by five cycles of denaturation at 98 °C for 3 min, annealing at 64 °C for 0.5 min, elongation at 72 °C for 6 min. After five cycles, the two separate reactions were combined and additional 20 cycles were carried out using the same temperature program followed by a final elongation step at 72 °C for 10 min. Methylated parental template DNA was digested with *Dpn*I. The reaction was stopped at 75 °C for 20 min. The resulting plasmid saturation libraries were designated as pET28a–iLOV–Q489K–V392X and pET28a–iLOV–Q489K–AG487X.

### Cloning and site-directed mutagenesis of CagFbFP

The mutation Q148K was introduced into the parental CagFbFP (22) encoding gene using two PCRs. In the first reaction, the gene fragment corresponding to amino acids 47 to 147 of the parent protein was amplified using pET11–CagFbFP as a template with 0.5 μM CagFbFP-fw: 5′-TAT ACA TAT GGC CAG CGG TAT GAT TGT T-3′ and CagFbFP-147-rev: 5′- CAC CAA CAA ATG CAA CAA CAT TGC C -3′ primers, 0.2 mM dNTPs, 1x KAPA2G buffer A, and KAPA2G Robust DNA Polymerase (Merck KGaA). Amplification was achieved using the following protocol: initial denaturation for 3 min at 95 °C, followed by 30 cycles of denaturation at 95 °C for 15 s, annealing at 60 °C for 15 s, and elongation at 72 °C for 15 s. The final elongation step at 72 °C was for 10 min. This gene fragment was excised from the gel and purified using the Cleanup Standard kit (Evrogen). A second extension PCR was performed using the purified fragment, an additional 0.02 μM oligonucleotide with mutation CagFbFP-Q148K-fw: 5′- GGC AAT GTT GTT GCA TTT GTT GGT GTT AAA ACA GAT GTT ACC GCA CAT CAT CAT C -3′ and 0.5 μM and primers CagFbFP-fw: 5′-TAT ACA TATG GCCA GCG GTA TGA TTG TT-3′ and CagFbFP-rev: 5′-AGC CGG ATC CTT AGT GAT GGT GAT G -3′, 0.2 mM dNTPs, 1x KAPA2G buffer A, and KAPA2G Robust DNA Polymerase (Merck KGaA). Amplification was achieved using the same protocol. The resulting gene with the Q148K mutation was excised from the gel and purified using the Cleanup Standard kit (Evrogen). The restriction reaction was carried out using *BamH*I and *Nde*I restriction enzymes. The resulting sticky ends were ligated to the vector pET11 using T4 DNA Ligase (Thermo Fisher Scientific). The mutation I52T was introduced into CagFbFP and CagFbFP–Q148K using PCR. The reaction mixture contained pET11–CagFbFP or pET11–CagFbFP–Q148K as a template with 0.5 μM CagFbFP–I52T_fw: 5′- CGG TAT GAC CGT TAC CGA TGC CGG-3′ and CagFbFP–I52T_rev: 5′-CGGTAACGGTCATACCGCTGGCC - 3′ primers, 0.2 mM dNTPs, 1x Phusion HF buffer, and Phusion High-Fidelity DNA polymerase (Thermo Fisher Scientific). Amplification was achieved using the following protocol: initial denaturation for 1 min at 98 °C, followed by 25 cycles of denaturation at 98 °C for 10 s, annealing at 55 °C for 0.5 min and elongation at 72 °C for 2.5 min. Final elongation step at 72 °C was for 10 min. Methylated parental template DNA was digested with *Dpn*I. The reaction was stopped at 80 °C for 5 min. Plasmids pET11–CagFbFP–I52T and pET11–CagFbFP–Q148K–I52T were transformed into *E. coli* DH10B cells plated on LB agar supplemented with ampicillin (100 μg/ml) and incubated over night at 37 °C. The plasmid DNA of the CagFbFP variants was isolated using the Plasmid Miniprep kit (Evrogen). The resulting mutated CagFbFP encoding DNA insert was subsequently sequenced (Evrogen) to verify the introduced amino acid exchange.

### Expression and high-throughput screening of the iLOV–Q489K–V392X site saturation library

*E. coli* BL21(DE3) cells were transformed with the pET28a–iLOV–Q489K–V392X and pET28a–iLOV–Q489K–G487X libraries, plated on LB agar supplemented with kanamycin (50 μg/μl), and incubated over night at 37 °C. To obtain a preculture, each well of a 96-deep-well plate, filled with 500-μl LB supplemented with kanamycin, was inoculated with a single colony. Three wells of each plate were used as controls, containing noninoculated medium, *E. coli* BL21(DE3) pET28a–iLOV, or pET28a–iLOV–Q489K. The deep well plates were then incubated at 37 °C and 800 rpm for 16 h. Subsequently, 500 μl of modified autoinduction media (AIM) (18, 37) was inoculated with the preculture using a microplate replicator and incubated at 37 °C and 800 rpm for 16 h. Cells were harvested by centrifugation at 2000 rpm for 30 min and lysed using BugBuster protein extraction reagent (Merck) following the instructions provided by the manufacturer. After centrifugation at 2000 rpm, 100 μl of the cell-free, protein-containing supernatant was transferred to a black 96-well microtiter plate (NUNC, Thermo Fisher Scientific) and emission spectra were recorded using an Infinite m1000 PRO microplate reader (Tecan; excitation wavelength, λ_ex_ = 440 nm; emission wavelength range, λ_em_ = 456–600 nm; slit width of 10 nm). Overall, five plates of a’ 93 clones (+3 controls), resulting in 465 individual clones per library, were screened. The plasmid DNA of the iLOV variants, identified in the screen to possess spectrally red-shifted fluorescence emission, was isolated from replica plates using the innuPREP Plasmid Mini Kit 2.0 (Analytik Jena). The resulting mutated iLOV encoding DNA insert was subsequently sequenced (Seqlab) to identify the introduced amino acid exchange.

### Expression and purification of iLOV and its variants

Verified plasmids (pET28a–iLOV, pET28a–iLOV–Q489K, pET28a–iLOV–Q489K–V392T) were transformed into *E. coli* CmpX131 (38). The corresponding proteins were produced and purified by immobilized metal-ion affinity chromatography (IMAC), as described previously (19). After IMAC purification, the imidazole-containing elution buffer was exchanged for the storage buffer (10 mM Tris/HCl, pH = 7.0, supplemented with 10 mM NaCl) by using a G25 Sephadex desalting column. Protein samples were concentrated using Macrosep advance centrifugal device (PALL; molecular weight cut-off = 3 kDa) and stored at 4 °C in the dark.

### Expression and purification of CagFbFP and its variants

Verified plasmids (pET11–CagFbFP, pET11–CagFbFP–Q148K, pET11–CagFbFP–I52T, pET11–CagFbFP–Q148K–I52T) were transformed into *E. coli* C41(DE3) or *E. coli* BL21(DE3). The corresponding proteins were produced and purified by IMAC, as described previously (22). After IMAC purification, the imidazole-containing elution buffer was exchanged for storage buffer (10 mM sodium phosphate, pH = 8.0, supplemented with 10 mM NaCl) by using size-exclusion chromatography on a Superdex 200 Increase 10/300GL column (GE Healthcare Life Sciences) or by using PD10 desalting columns (GE Healthcare Life Sciences). Protein samples were concentrated using Amicon Ultra-4 Centrifugal Filter Unit (Merck KGaA; molecular weight cut-off = 10 kDa) and stored at 4 °C in the dark.

### Fluorescence spectroscopy

Fluorescence emission and excitation spectra were recorded using a QuantaMaster 40 fluorescence spectrophotometer (Photon Technology International) as described previously by Wingen *et al.* (18). Absorbance spectra were recorded using a Cary 60 UV/Vis spectrophotometer (Agilent Technologies) equipped with a Peltier thermostatted single cell holder. Fluorescence quantum yields were determined using a QuantaMaster 40 fluorescence spectrophotometer (Photon Technology International) equipped with an integrating sphere as described (18). All measurements were carried out in the storage buffer (10 mM Tris/HCl, pH = 7.0, supplemented with 10 mM NaCl; iLOV and its variants, or in 10 mM sodium phosphate, pH = 8.0, supplemented with 10 mM NaCl; CagFbFP and its variants).

### Fluorescence lifetime measurements

The time-resolved detection of the fluorescence intensity decay of all FbFPs was performed using a Fluotime100 fluorescence spectrophotometer (PicoQuant) based on a picoHarp300 unit by using a pulsed diode laser (Laser PicoQuant LDH-C440; emission: 440 nm; pulse width: 50 ps; used repetition frequency: 20 MHz) as an excitation source. Fluorescence decay curves as a function of time (t) were measured by time-correlated single-photon counting that enables the determination of fluorescence decay components with fluorescence lifetimes greater than 100 ps (39, 40). Decay curves were analyzed by iterative reconvolution of the instrument response function, IRF(t), with an exponential model function, M(t), using the FluoFit software (version 4.5.3.0; PicoQuant) applying Equations 1 and 2:

(1) I(t)=IRF(t)×M(t)

(2) $$M\left(t\right)=\;\sum \alpha_{i}\cdot \exp\left(-\frac{t}{\tau_{i}}\right);n=1,2$$

τ_i_ is the characteristic lifetime, and α_i_ is the respective intensity. The average fluorescence lifetime, τ_fl,ave_, was calculated using Equation 3:

(3) $$\tau_{fl,ave}=\;\sum \frac{\alpha_{i}\cdot \tau_{i}}{\sum \;\alpha_{i}}$$

### Protein crystallization

The purified iLOV–Q489K protein (in 10 mM Tris/HCl, pH = 7.0, supplemented with 10 mM NaCl) was concentrated to 26 mg/ml using Vivaspin centrifugal concentrators (Sartorius; 20-kDa molecular weight cut-off). Crystallization setups were performed using the sitting drop vapor diffusion method (1 μl of protein and 1 μl of the reservoir solution) and stored at 19 °C in the dark. Tetragonal crystals grew within 6 weeks against 0.1 M sodium acetate (pH 4.6) containing 25% (w/v) PEG 1000. The purified CagFbFP variants (in 10 mM sodium phosphate, pH = 8.0, supplemented with 10 mM NaCl) were concentrated to 30 mg/ml using the Amicon Ultra-4 Centrifugal Filter Unit (Merck KGaA; molecular weight cut-off = 10 kDa). Crystallization setups were performed using the sitting drop vapor diffusion method (150 nl of protein and 100 nl of the reservoir solution) and stored at 22 °C in the dark. CagFbFP–Q148K crystals were obtained using the precipitant solution containing 0.1 M 2-(N-morpholino)ethanesulfonic acid monohydrate, pH 6.5, and 12% w/v PEG 20000. Morpholine-free CagFbFP–Q148K structure was obtained using 0.5 M ammonium sulfate, 1 M lithium sulfate monohydrate, 0.1 M sodium citrate tribasic dihydrate, pH 5.6. CagFbFP–I52T crystals were obtained using the precipitant solution containing 0.2 M lithium sulfate, 0.1 M Bis-Tris, pH 5.5, and 25% PEG 3350. CagFbFP–I52T–Q148K crystals were obtained using the precipitant solution containing 0.1 M citrate, pH 5.0, and 20% PEG 6000.

### Data collection and structure determination

iLOV–Q489K crystals grew in high PEG concentrations and were cryo-cooled directly at 100 K. X-ray diffraction data were recorded at the beamline ID29 (European Synchrotron Radiation Facility (41)) tuned to a wavelength of 0.91 Å on a PILATUS 6M-F detector (Dectris Ltd). Data collection strategy was based on calculations using the program BEST that accounts for radiation damage and symmetry (42). Data processing was carried out using the program XDS (43) and AIMLESS (part of the CCP4 package (44)). The initial phases were obtained by molecular replacement using MOLREP (44), and the search model was generated from the crystal structure of iLOV (PDB ID: 4EES). Analysis of Matthews coefficient (44) suggested one molecule per asymmetric unit, with Matthews coefficient of 1.82 Å^3^/Da and the solvent content of 32.6%. The model was further improved with several cycles of refinement using the PHENIX package (45) and manual rebuilding using the program COOT (46). Data collection and refinement statistics are listed in Table S1.

CagFbFP crystals were cryoprotected by adding the precipitant solution (see above) supplemented with 20% glycerol and were cryo-cooled at 100 K. X-ray diffraction data for the different CagFbFP crystals was recorded at various beamlines (see Table S1 for details). Data collection strategy was based on calculations using the program BEST that accounts for radiation damage and symmetry (42). Data processing was carried out using the program XDS (43) and AIMLESS (part of the CCP4 package (44)). The dataset 6YX6 (Table S1) revealed strong anisotropy and was processed using STARANISO (47) using the following criterion for determining the diffraction-limit surface, <I_mean_/σI_mean_>, of more than 2. The initial phases were obtained by molecular replacement using MOLREP (44), and the search model was generated from the crystal structure of CagFbFP (PDB ID: 6RHF). The model was manually refined using the programs COOT (46) and REFMAC5 (48). Data collection and refinement statistics are listed in Table S1.

### QM/MM calculations

QM/MM optimizations and spectral calculations were performed with the program package ChemShell v3.5 (www.chemshell.org). We used Turbomole 6.3 (49) for the QM part and DL_POLY (50) as driver of the Amber (51) force field for the MM part. Before QM/MM calculations, force field–based energy minimization was carried out using Amber14 program (52) with the Amber ff99SB (53, 54) all-atom force field for proteins and general Amber force field (51) for FMN and the TIP3P model for water (55). We used the available atomic charges and force-field parameters for FMN reported in our previous work (19). The initial coordinates for simulations were taken from X-ray structure of iLOV (PDB ID: 4EES; (27)), iLOV–Q489K (PDB ID: 7ABY), and parental CagFbFP (PDB ID: 6RHF; (22)), CagFbFP–I52T–Q148K (PDB ID: 7AB7). iLOV proteins contain one iLOV molecule per asymmetric unit and thus represents a monomeric structure, whereas CagFbFP variants have two CagLOV molecules per asymmetric unit forming a homodimeric structure. The initial coordinates of chain A of CagFbFP–Q148K–I52T structure were used for all simulations. The protonation states of titratable residues at pH 7 were assigned on the basis of pKa calculations using the PROPKA 3.1 program (56) and visual inspection. In simulations, all charged residues were kept at their standard protonation states. Side chains of Asn and Gln residues were checked for possible flipping. To neutralize the systems, solvent water molecules that were at least 5.5 Å away from any protein atoms were replaced by Na^+^. Hydrogen atoms were added using the tleap module of AmberTools14 (52). The protein was solvated in a water box of 12 Å radius centered at the center of mass. Crystal water molecules were kept. To calculate the electrostatic interactions, a cutoff of 10 Å was used. The total size of the simulation systems was ∼27,000 atoms, including ∼8500 TIP3P (55) water molecules. The solvent and the ions followed by the whole system were subjected to minimization using 10,000 steps of steepest descent followed by 3000 steps of conjugate-gradient minimization. The minimized structures from the energy minimization runs were chosen as starting structures for further QM/MM calculations. For the QM part of the QM/MM calculation, time-dependent density functional with B3LYP density functional (57, 58, 59) and the SVP basis set (57, 58, 59) from the Turbomole basis set library has been chosen to obtain vertical excitation and emission energies. Reliable excitation energies have already been obtained by this combination of method and basis set for electronic states of the FMN in the LOV domain (19). Geometries of the ground state (S_0_) and first singlet excited state (S_1_) were optimized at QM/MM DFT/AMBER level of theory. We have applied the additive QM/MM scheme with electrostatic embedding. The charge-shift scheme (60) and hydrogen link atoms were used to handle the QM-MM boundary region. During the QM/MM structure optimizations which were carried out using the DL-FIND (61) optimizer module of ChemShell, we relaxed all fragments (amino acids, FMN, water, counter ions) that lie within 10 Å of the FMN chromophore. Everything beyond this selection was kept frozen during QM/MM structure optimizations. The QM region consists of the FMN chromophore, whereas the covalent bond (between C13 and C15 for FMN) across the QM-MM boundary was capped with hydrogen link atoms. VMD (62) and AmberTools14 (52) were used for molecular visualizations and analysis of simulations.

### Microscopy

*E. coli* C41(DE3) cells were grown in a shaker at 37 °C. Expression was induced using 1 mM IPTG when the optical density of 0.6 was reached. Expression of CagFbFP–Q148K and CagFbFP–Q148K–I52T was continued for 100 min and 5 h, respectively. The cells were harvested by centrifugation at 4000*g* for 3 min. The pellets were resuspended in the PBS and diluted to the optical density of approximately 0.5 and placed on cover slips for imaging. The lambda stacks (459–557 nm, 9 nm step) were obtained using the LSM780 system (Carl Zeiss) with 100× objective (oil immersion, numerical aperture = 1.46). Fluorescence was excited by pulsed 900-nm InSight DeepSee laser (20 mW at the objective). Images were obtained with the resolution of 2048 × 2048 pixels, pixel dwell time of 3.1 μs, and 4× line averaging. Afterward, the images were averaged using 5 pixel moving average to further reduce spectral variation between neighboring pixels (caused by noise). The linear unmixing was performed with the ZEN software (Carl Zeiss) utilizing the fluorescence emission spectra obtained from images of cells expressing only either CaFbFP–Q148K or CagFbFP–Q148K–I52T.

## Data availability

All data described in the article are contained within the article and Supporting information. Protein crystal structures have been deposited under the following accession numbers (see also Table S1 for details): iLOV–Q489K: PDB ID: 7ABY; CagFbFP–Q148K: PDB ID: 6YX4, 6YX6, and 6YXB; CagFbFP–I52T: PDB ID: 7AB6; CagFbFP–I52T–Q148K: PDB ID: 7AB7.

## Supporting information

This article contains supporting information (19, 63, 64).

## Conflict of interest

The authors declare that they have no conflicts of interest with the contents of this article.

## Supporting information

Suppleemntal Figures S1–S5 and Tables S1–S3
