# Supplementary material for: The molecular basis of spectral tuning in blue- and red-shifted flavin-binding fluorescent proteins
Source: J Biol Chem. 2021 Apr 19;296:100662. doi: 10.1016/j.jbc.2021.100662 (PMC8131319; doi:10.1016/j.jbc.2021.100662)
Supplement: Suppleemntal Figures S1–S5 and Tables S1–S3 [file mmc1.pdf]

SUPPORTING INFORMATION

The molecular basis of spectral tuning in blue- and red-shifted flavin-binding fluorescent proteins

Katrin Röllen[1], Joachim Granzin[2,3], Alina Remeeva[4], Mehdi D. Davari[5], Thomas Gensch[6], Vera V. Nazarenko[4], Kirill Kovalev[2,3,4,8,9], Andrey Bogorodskiy[4], Valentin Borshchevskiy[4], Stefanie Hemmer[1,10], Ulrich Schwaneberg[5,7], Valentin Gordeliy[2,3,4,8], Karl-Erich Jaeger[1,10], Renu Batra-Safferling[2,3], Ivan Gushchin[4*], Ulrich Krauss[1,10*]

[1]Institut für Molekulare Enzymtechnologie, Heinrich-Heine-Universität Düsseldorf, Forschungszentrum Jülich GmbH, D-52425 Jülich, Germany

[2]IBI-7: Structural Biochemistry, Forschungszentrum Jülich GmbH, D-52425 Jülich, Germany

[3]JuStruct: Jülich Center for Structural Biology, Forschungszentrum Jülich, 52428 Jülich, Germany

[4]Research Center for Molecular Mechanisms of Aging and Age-Related Diseases, Moscow Institute of Physics and Technology, 141701 Dolgoprudny, Russia

[5]Institute of Biotechnology, RWTH Aachen University, Worringer Weg 3, D-52074 Aachen, Germany

[6]IBI-1: Molecular and Cellular Physiology, Forschungszentrum Jülich GmbH, D-52425 Jülich, Germany

[7]DWI-Leibniz Institute for Interactive Materials, Forckenbeckstraße 50, D-52074, Aachen, Germany

[8]Institut de Biologie Structurale Jean-Pierre Ebel, Université Grenoble Alpes-Commissariat à l'Energie Atomique et aux Energies Alternatives–CNRS, 38044 Grenoble, France

[9]Institute of Crystallography, RWTH Aachen University, 52062 Aachen, Germany

[10]IBG-1: Biotechnology, Forschungszentrum Jülich GmbH, D-52425 Jülich, Germany

Corresponding authors:

Ulrich Krauss, Institut für Molekulare Enzymtechnologie, Heinrich-Heine Universität Düsseldorf, Forschungszentrum Jülich GmbH, D-52425 Jülich, Germany, Phone: +49 2461-61-2939, Email: u.krauss@fz-juelich.de

Ivan Gushchin, Research Center for Molecular Mechanisms of Aging and Age-Related Diseases, Moscow Institute of Physics and Technology, 141701 Dolgoprudny, Russia, Phone: +7-965-428-22-24, Email: ivan.gushchin@phystech.edu

Table of contents

Item	Content	Page
**Supporting Figures and Tables**		S3-S10
Table S1a	Data collection and refinement statistics	S3
Figure S1	Structure of iLOV-Q489K and CagFbFP-Q148K.	S4
Table S2	Screening results - V392X and G487X libraries of iLOV-Q489K	S5
Figure S2	Screening results – fluorescence emission spectra of the most promising red-shifted iLOV-Q489K variants	S6
Figure S3	Structure of CagFbFP-I52T-Q148K	S7
Figure S4	Fluorescence lifetime data for all iLOV variants	S8
Figure S5	Fluorescence lifetime data for all CagFbFP variants	S9
Table S3	QM/MM calculation of iLOV and CagFbFP spectral properties	S10
**Supporting References**		S11

Table S1a: Data collection and refinement statistics. Statistics for the highest-resolution shell are shown in parentheses.

Variant, PDB ID	iLOV-Q489K 7ABY	CagFbFP-Q148K 6YX4	CagFbFP-Q148K 6YX6	CagFbFP-Q148K 6YXB	CagFbFP-I52T 7AB6	CagFbFP-I52T Q148K 7AB7
Beamline/Detector	ESRF ID29 DECTRIS Pilatus 6M-F 2017-11-27	ESRF ID29 DECTRIS Pilatus 6M-F 2018-02-09	ESRF ID29 DECTRIS Pilatus 6M-F 2018-02-09	ESRF ID29 DECTRIS Pilatus 6M-F 2018-02-09	SLS X06SA DECTRIS EIGER X 16M 2019-03-24	PETRA III P13 (MX1) DECTRIS PILATUS 6M 2019-05-20
Wavelength λ(Å)	0.9194	0.9737	0.9737	0.9737	0.999987	0.9762
Resolution range (Å)	43.93-1.45 (1.48-1.45)	39.90-1.36 (1.38-1.36)	39.99-1.50 (1.54-1.50)	110.54-1.50 (1.53-1.50)	48.49-1.90 (1.94-1.90)	53.08-1.80 (1.84-1.80)
Space group	$P\ 4_3\ 2_1\ 2$	$P\ 2_1 2_1 2$	$P\ 2_1 2_1 2$	$P\ 2_1$	$P\ 2_1\ 2_1\ 2$	$P\ 2_1\ 2_1\ 2$
Unit cell a, b, c (Å); α,β,γ=90 (°)	40.76, 40.76, 131.79	57.66, 110.56, 39.01	57.77, 110.84, 39.20	39.10, 110.54, 56.94 β = 91.07	53.866, 110.784, 39.094	53.083, 111.992, 39.031
Total reflections	186700	314069	199987	228720	141766 (9259)	72891 (4318)
Unique reflections	20710 (1030)	53232 (2525)	35712 (1786)	76240 (3706)	19058 (1199)	22158 (1293)
Multiplicity	9.0 (9.6)	5.9 (5.8)	5.6 (5.6)	3.0 (3.0)	7.4 (7.7)	3.3 (3.3)
Completeness (%)	100.0 (100.0)	97.6 (94.4)	86.8 (59.9)*	98.9 (98.1)	99.6 (100.0)	99.5 (99.8)
Mean I/sigma(I)	14.3 (2.1)	15.3 (2.0)	14.8 (2.9)	10.6 (2.6)	9.3 (2.2)	7.1 (1.5)
Wilson B-factor (Å^2)	16.6	14.1	13.3	9.3	13.2	17.9
R-merge	0.070 (1.107)	0.046 (0.696)	0.082 (0.575)	0.062 (0.392)	0.141 (0.832)	0.068 (0.509)
R-meas	0.074 (1.170)	0.056 (0.843)	0.090 (0.635)	0.087 (0.538)	0.163 (0.955)	0.091 (0.680)
Mn(I) half-set correlation CC(1/2)	0.999 (0.775)	0.999 (0.799)	0.999 (0.881)	0.997 (0.676)	0.996 (0.824)	0.996 (0.745)
**Refinement**						
Resolution range (Å)	38.94-1.45 (1.502-1.45)	39.94-1.36 (1.395-1.359)	39.99-1.50 (1.539-1.530)	56.95-1.50 (1.539-1.530)	48.49-1.90 (1.949-1.90)	48.01-1.80 (1.847-1.80)
R-work	0.1563 (0.1750)	0.1401 (0.1980)	0.1740 (0.241)	0.1835 (0.269)	0.1797 (0.2350)	0.1587 (0.2330)
R-free	0.1847 (0.2450)	0.1721 (0.2130)	0.2019 (0.268)	0.2028 (0.275)	0.2131 (0.2210)	0.1947 (0.2940)
coordinate error (max.-likelihood based)	0.12	0.031	0.050	0.050	0.09	0.09
Number of non-hydrogen atoms	1037	2017	2068	3873	1954	2001
macromolecules	885	1615	1645	3253	1692	1741
ligands	46	62	62	124	62	62
water	106	340 (solvent)	361 (solvent)	496 (solvent)	200 (solvent)	198 (solvent)
Protein residues	108	206	210	416	224	233
RMS (bonds)	0.010	0.007	0.005	0.005	0.003	0.011
RMS (angles)	1.12	1.4	1.3	1.3	1.22	1.72
Ramachandran favored (%)	100.0	97	97	98	99.55	99.13
Ramachandran allowed (%)	0.0	3	2.5	2	0.45	0.87
Clashscore	7.74	4	7	5	5	5
Average B-factor (Å^2)	25.17	23.15	16.88	14.67	21.65	23.37
macromolecules (Å^2)	24.08	21.0	14.3	13.2	20.90	22.49
ligands (Å^2)	19.8	15.4	8.1	8.4	16.68	15.29
solvent (Å^2)	36.57	34.6	30.2	25.9	29.58	33.63

* Ellipsoidal completeness is 96.2 (98.3)

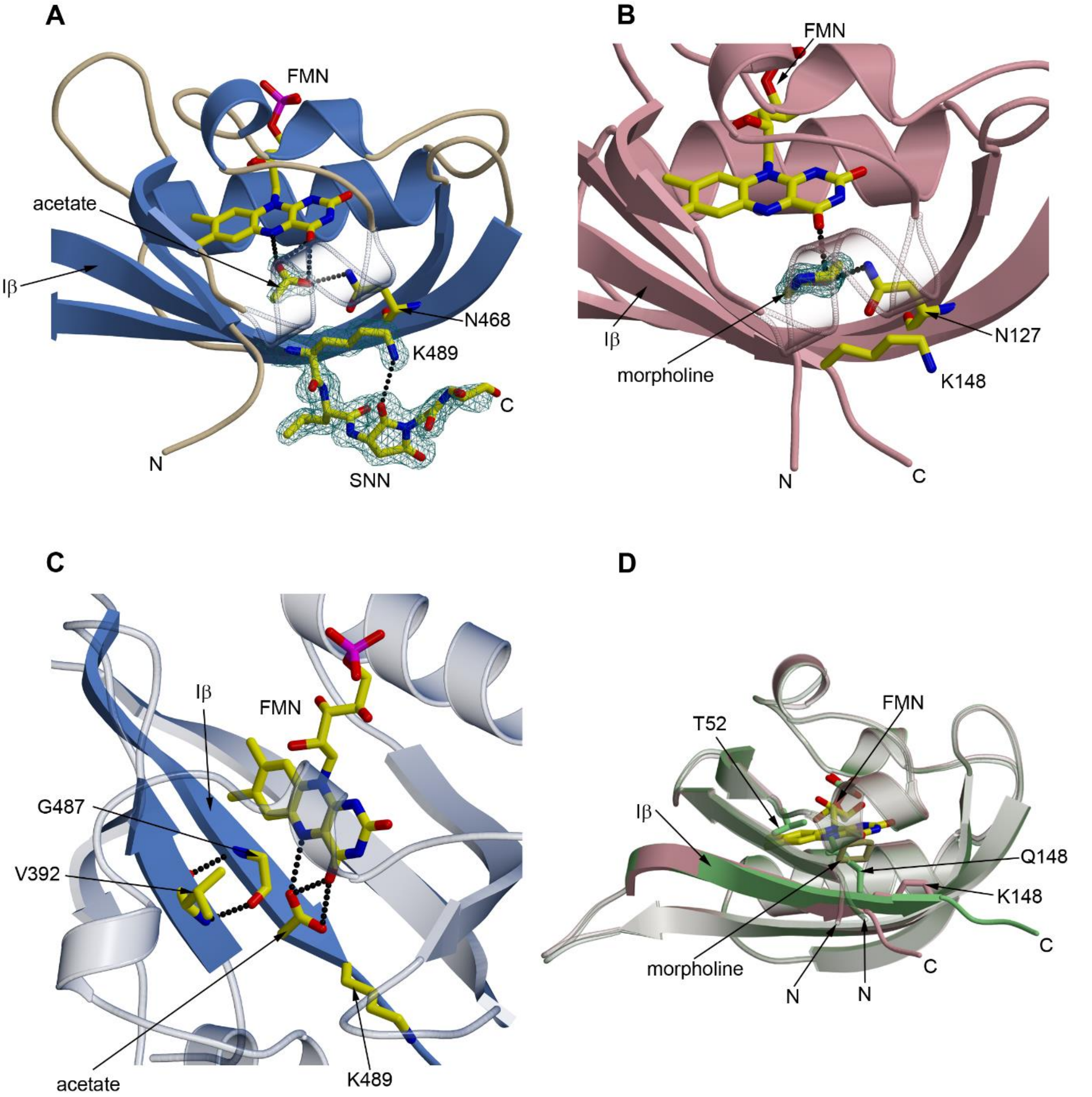

Figure S1: Structure of iLOV-Q489K and CagFbFP-Q148K. (A) iLOV-Q489K mutant with the electron density map for the acetate ion and the C-terminus with its modification of the N491 and G492 (see description of Fig. 2b). The 2mFo-DFc difference map is shown at 1.0 rms. (B) 2mFo-DFc difference map at 1.0 rms of the morpholine molecule of CagFbFP-Q148K (highest resolution structure, PDB-ID: 6YX4). Dashed lines represent hydrogen bonds with a donor-acceptor distance of ≤ 3.2 Å. More information can be found in the legend of Figure 2D in the main manuscript. (C) Structure of iLOV-Q489K highlighting the position of the introduced K489 in relation to the amino acids V392 and G487, which were targeted by saturation mutagenesis. The two residues are located directly opposite each other in two anti-parallel β-strands and have the typical backbone hydrogen bonds. (D) Superposition of CagFbFP-I52T (light coral) with CagFbFP-Q148K (plum), demonstrating again that the Q148K point mutation exclusively affects the C-terminal backbone conformation.

Table S2: Screening results - emission maxima of selected iLOV-Q489K variants identified in the V392X and G487X libraries. Additionally, the reference emission maxima for parental iLOV and iLOV-Q489K are shown.

iLOV-Q489K-V392X library			iLOV-Q489K-G487X library		
Clone	Emission maxima (nm)	V392 substitution to	Clone	Emission maximum (nm)	G487 substitution to
iLOV	499/524	-	iLOV	499	
Q489K	490/511	-	Q489K	490	
B1-E9	510/532	Thr	B2-A6	502	Ser
B1-H5	510/526	Thr	**B2-D8**	**502**	**Ser**
B2-A3	504/527	Thr			
B2-E8	510/526	Thr			
B2-E10	503/514	Cys			
B2-F12	502/517	Thr			
B3-B12	504/529	Thr			
B3-E5	505/529	Thr			
B4-D11	495/514	Ala			
B4-F12	507/530	Thr			
B4-G11	507/527	Thr			
B5-B3	507/528	Thr			
**B5-B12**	**507/529**	**Thr**			
B5-C3	504/532	Thr			
B2-C5	504/529	Thr			
B5-F2	504/529	Thr			

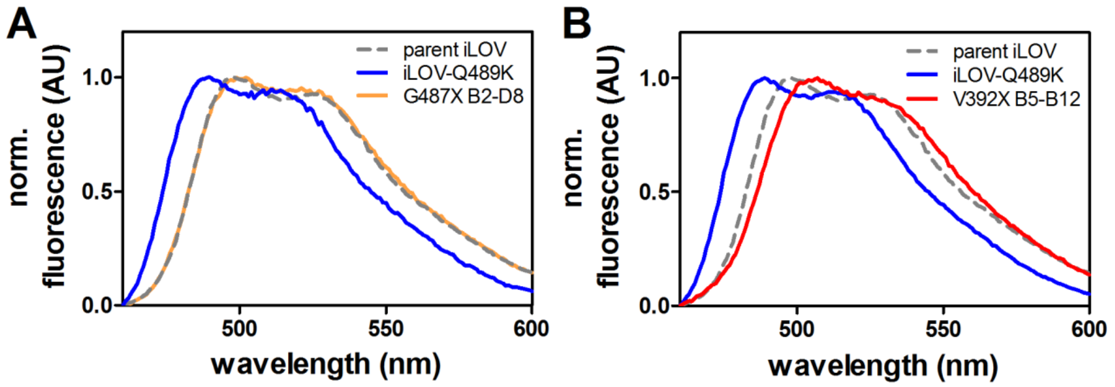

Figure S2: Screening results – fluorescence emission spectra of the most promising red-shifted iLOV-Q489K variants identified in the G487X (A) and V392X (B) libraries. Variants, with red-shifted fluorescence emission were identified from a library of 465 clones per library. Emission spectra were recorded using a Tecan Infinite m1000 fluorescence plate reader, as described in the Materials and Methods section. Spectra were normalized to yield equal values at the main emission peak maximum. As reference, each panel also contains the emission spectrum of parental iLOV (grey dashed line) and iLOV-Q489K (blue solid line). Spectra normalized to the corresponding maximum.

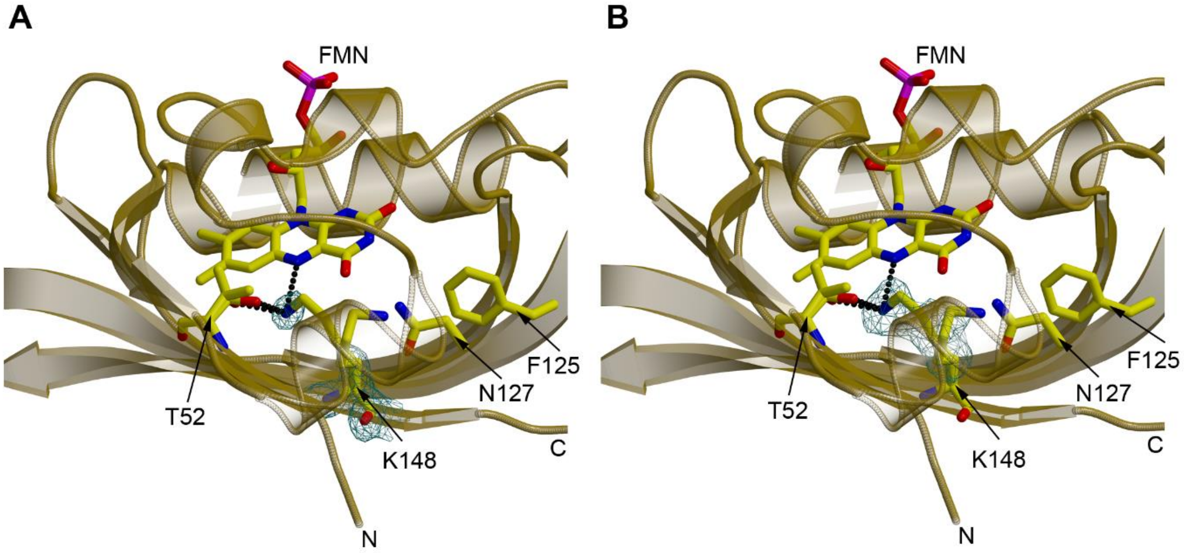

Figure S3: Structure of CagFbFP-I52T-Q148K with two differently calculated electron density maps around the K148. Additionally, the side chains of N127 and F125 are depicted. A) 2mFo-DFc difference map at 1.0 rms illustrating the high flexibility of Lys 148, only the conformation that interacts with the Thr 52 via hydrogen bonds seems to be prominent. (B) Polder electron density map at 3.5 rms. This map is based on the removal of the bulk solvent area around the excluded region, here the lysine was excluded from the calculation. Please note that no bias-removal strategies were applied. The map hints at two rotamer conformations for K148.

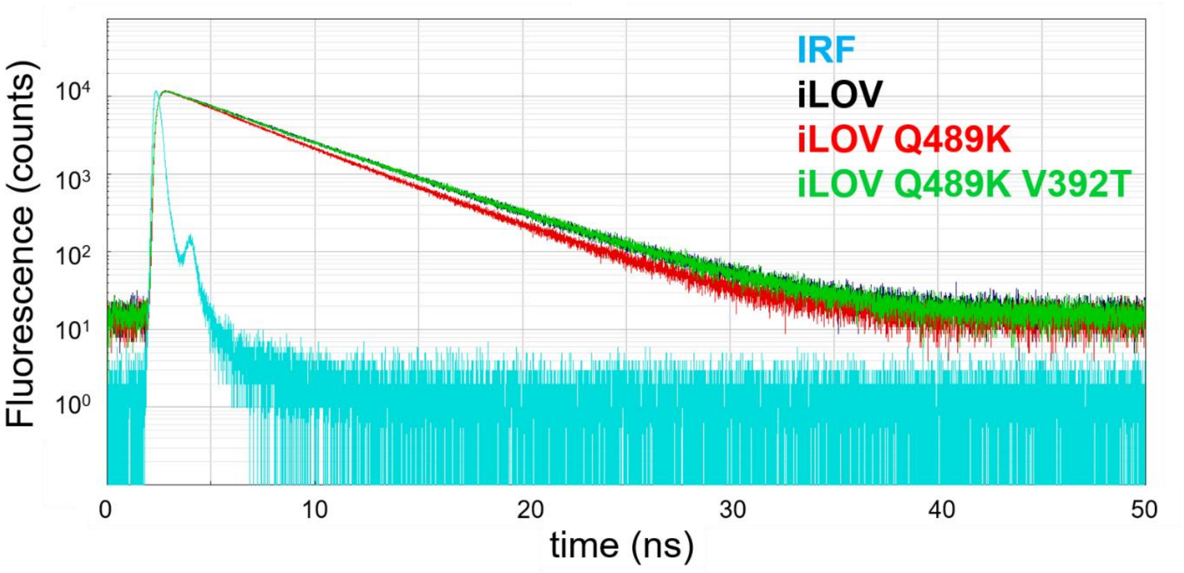

Figure S4: Time course of fluorescence intensity after ps-pulsed excitation of iLOV (black), iLOV-Q489K (red) and iLOV-Q489K-V392T (green). The instrument response function (IRF; cyan) illustrates the temporal resolution of the setup. The mean values of the fit parameters are given below:

**protein**	**a_1 (%)**	**τ_1 (ns)**	**a_2 (%)**	**τ_2 (ns)**
iLOV	17	2.60	83	4.83
iLOV-Q489K	18	2.29	82	4.50
iLOV-Q489K-V392T	16	2.10	84	4.82

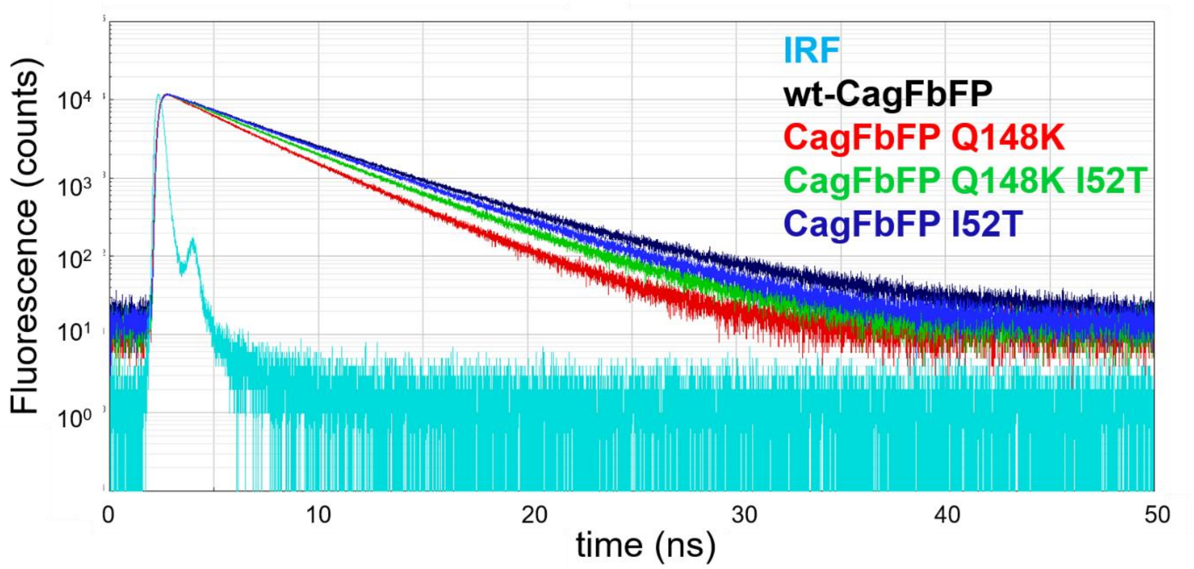

Figure S5: Time course of fluorescence intensity after ps-pulsed excitation of CagFbFP (black), CagFbFP-Q148K (red), CagFbFP-Q148K-I52T (green) and CagFbFP-I52T. The instrument response function (IRF; cyan) illustrates the temporal resolution of the setup. The mean values of the fit parameters are given below:

**protein**	**a_1 (%)**	**τ_1 (ns)**	**a_2 (%)**	**τ_2 (ns)**
CagFbFP	52	3.41	48	5.77
CagFbFP-Q148K	45	2.44	55	3.97
CagFbFP-Q148K-I52T	34	2.60	66	4.47
CagFbFP-I52T	51	3.51	49	5.40

QM/MM calculation of iLOV and CagFbFP spectral properties

Note that a direct comparison between QM/MM calculated excitation and fluorescence-emission wavelengths and the corresponding experimental data is difficult, because QM/MM methods calculate vertical transition energies for given electronic transition. Those, however, do not directly compare to the experimentally observed excitation and fluorescence emission maxima due to vibronic effects (1-3).

Table S3: QM/MM derived excitation and fluorescence emission wavelengths derived from vertical transition energies of the relevant electronic transition. Calculations were performed for two T52 conformation (A, B) present in the CagFbFP-Q148K-I52T structure.

	$\lambda_{max\text{-}excitation}$ (nm)	shift (nm)	$\lambda_{max\text{-}emission}$ (nm)	shift (nm)
iLOV	415	-	490	-
iLOV-Q489K	352	-63	393	-96
CagFbFP	417	-	491	-
CagFbFP-Q148K-I52T (A)	443	26	536	45
CagFbFP-Q148K-I52T (B)	442	26	540	49

Supporting References

1. Avila Ferrer, F. J., Cerezo, J., Stendardo, E., Improta, R., and Santoro, F. (2013) Insights for an Accurate Comparison of Computational Data to Experimental Absorption and Emission Spectra: Beyond the Vertical Transition Approximation. *J Chem Theory Comput* **9**, 2072-2082
2. Davari, M. D., Kopka, B., Wingen, M., Bocola, M., Drepper, T., Jaeger, K. E., Schwaneberg, U., and Krauss, U. (2016) Photophysics of the LOV-based fluorescent protein variant iLOV-Q489K determined by simulation and experiment. *J. Phys. Chem. B* **120**, 3344-3352
3. Klaumünzer, B., Kroner, D., and Saalfrank, P. (2010) (TD-)DFT calculation of vibrational and vibronic spectra of riboflavin in solution. *J Phys Chem B* **114**, 10826-10834
